# Longitudinal associations between loneliness, social isolation and cardiovascular events

**DOI:** 10.1136/heartjnl-2020-316614

**Published:** 2020-05-27

**Authors:** Feifei Bu, Paola Zaninotto, Daisy Fancourt

**Affiliations:** 1 Behavioural Science and Health, University College London, London, UK; 2 Epidemiology and Public Health, University College London, London, UK

**Keywords:** cardiac risk factors and prevention, epidemiology

## Abstract

**Objective:**

This study aimed to examine the association between loneliness, social isolation and cardiovascular disease (CVD), looking at both self-reported CVD diagnosis and CVD-related hospital admissions.

**Methods:**

Data were derived from the English Longitudinal Study of Ageing linked with administrative hospital records and mortality registry data. The analytical sample size was 5850 for the analysis of self-reported CVD and 4587 of CVD derived from hospital records, with a follow-up up to 9.6 years. Data were analysed using survival analysis, accounting for competing risks events.

**Results:**

The mean age was 64 years (SD 8.3). About 44%–45% were men. Within the follow-up, 17% participants reported having newly diagnosed CVD and 16% had a CVD-related hospital admission. We found that loneliness was associated with an increased risk of CVD events independent of potential confounders and risk factors. The hazard of people with the highest level of loneliness was about 30% higher for onset CVD diagnosis (HR: 1.05, 95% CI: 1.01 to 1.09) and 48% higher for CVD-related hospital admissions (HR: 1.08, 95% CI: 1.03 to 1.14), compared with the least lonely. There was little evidence that social isolation was independently associated with the risk of either CVD diagnosis or admission.

**Conclusions:**

Our findings provided strong evidence for the relationship between loneliness and cardiovascular events. Loneliness should be considered as a psychosocial risk factor for CVD in both research and interventions for cardiovascular prevention.

## Introduction

Cardiovascular diseases (CVD) is a major health problem globally and in the UK. According to the British Heart Foundation, there are 7.4 million people living with CVD in the UK.^1^ CVD is the second leading cause in the UK which accounted for 27% of all death.^2^ CVD, in particular stroke, is also a major contributor of acquired adult disability. It was reported from 55% to 77% of stroke survivors were severely disabled or required assistance with daily activities.^3^ It imposed a major financial burden on public expenditure, costing the UK £9 billion for healthcare and another £4 billion for non-healthcare.^4^ The National Health Service (NHS) long-term plan set CVD as one of its clinical priorities, setting an ambition to prevent CVD cases over the next 10 years.^5^

A large number of studies have identified a wide range of risk factors that are associated with CVD. These can be classified into two groups: non-modifiable and modifiable risk factors. The former includes demographic characteristics (eg, age, gender, ethnicity) and family history.^6^ The latter can be further broken down into three categories: (1) biological conditions, such as obesity, diabetes, hypertension and dyslipidaemia,^7 8^ (2) psychosocial factors, such as stress, anxiety and depression,^7 9^ and (3) behavioural measures, including sleep, physical activity, drinking, smoking and diet.^7 10^

Over the past few years, there has been growing interest in loneliness as a psychosocial risk factor and social isolation as a behavioural risk factor. Previous research has identified loneliness and social isolation as risk factors for all-cause and CVD-specific mortality.^11 12^ However, there is less evidence as to whether loneliness and social isolation are risk factors for CVD *incidence*. A few studies have focused specifically on CHD, but results are mixed.^13–15^ A recent meta-analysis of longitudinal studies showed that poor social relationships were associated with a 29% increase in the risk of CHD and 32% for stroke.^13^ However, Hakulinen *et al* 14 found that the relationship between loneliness, social isolation and both heart attack and stroke was attenuated after considering other risk factors. A recent study looking at CVD more broadly found that living alone (one aspect of social isolation) was associated with a higher risk of CVD.^16^ Another study, however, found that loneliness but not social isolation was associated with a 27% increase in the risk of CVD independently from other risk factors.^15^

There is very limited research looking beyond CHD and stroke at a broader range of CVD, and it remains inconclusive whether the relationship of loneliness and social isolation to CVD is similar. Furthermore, most studies have focused on self-reported diagnoses, but using routinely collected hospital records could provide more objective data on CVD incidence and health service utilisation. Therefore, the present study aimed to extend the research on loneliness, social isolation and CVD using data from the English Longitudinal Study of Ageing (ELSA) linked to Hospital Episodes Statistics (HES).

## Data and method

### Data and variables

Data came from ELSA, a nationally representative panel study of people aged 50 years or over and their partners, living in private households in England. The original sample was drawn from participants from the Health Survey in England (HSE) in 1998, 1999 and 2001. The first wave of ELSA took place in 2002/2003, with biennial follow-ups. Data collection is carried out through face-to-face interviews, self-completion questionnaires and nurse visits (every 4 years). We used wave 4 (2008/9) as our baseline because some variables of interest were not measured at earlier waves. We restricted participants to core ELSA members (89%). Furthermore, we excluded participants who did not return the self-completion questionnaire where social isolation was measured (16%). The analytical sample size consisted of 8310 participants.

#### Cardiovascular disease

**Figure 1:**
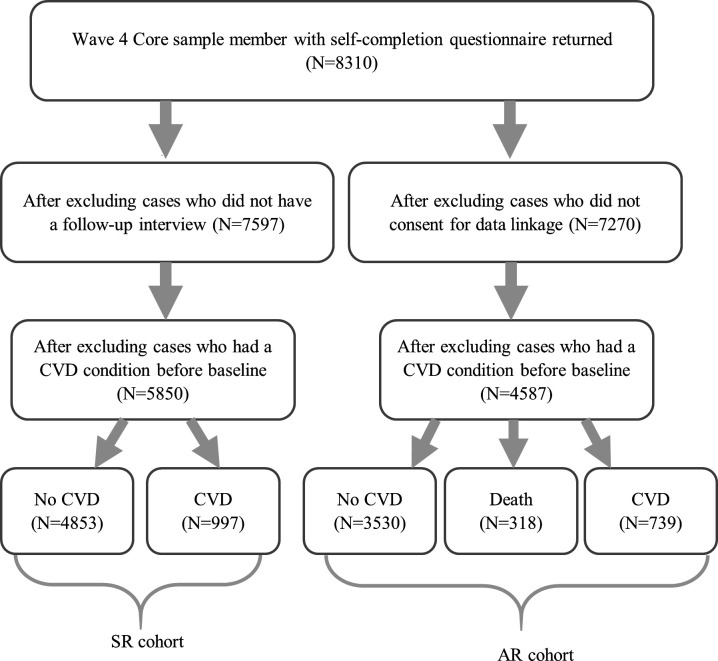
Sample selection diagram for the self-reported cohort and the hospital admission cohort.

In this study, CVD events were identified from two sources: self-reported doctor-diagnosed conditions from ELSA (SR-CVD) and administrative records (AR-CVD). During the interview participants were asked whether they had any of the following CVD diagnosis, including angina, heart attack, congestive heart failure, heart murmur, abnormal heart rhythm, stroke and other heart disease. The corresponding dates of diagnoses were attempted, but where these could not be recalled by participants, the date of interview was used as a proxy. In this study, we considered only CVD diagnosed after the baseline, so participants who did not have a follow-up interview (n=713, 9%) were excluded from the analysis (figure 1). Furthermore, we excluded participants who had reported any CVD condition at or before the baseline (n=1747, 23%). This left us a sample of 5850 individuals for the analysis of SR-CVD. Around 17% of them developed onset CVD within the study period.

CVD is a broad term, covering a wide range of conditions affecting the heart or blood vessels. The list of conditions that were asked in ELSA (or through any self-report) is not exhaustive. Therefore, as a comparative approach, we looked at CVD-related hospital admissions by linking ELSA data with the Admitted Patient Care (APC) data from the NHS HES. In addition, we also included a small number of participants who died from CVD but did not have a CVD admission as we assumed that the CVD event which led to their death would have been serious enough to have led to a hospital admission. Information on death was obtained from the UK NHS mortality registry. In this study, AR-CVDs were defined as hospital admissions with a primary diagnosis as CVD or death from CVD (without prior CVD admissions). Diagnoses in the APC data and cause of death in the mortality data were both coded using the International Statistical Classification of Disease and Related Health Problems 10th Revision (ICD-10). CVDs corresponded to the ICD-10 codes from I00 to I99, providing a much more comprehensive index than through self-reports. The APC data were available from February 1997 to January 2018, allowing us to follow-up participants from the baseline (2008/9) up to 9.6 years. As shown in figure 1, for the analysis of AR-CVDs, we excluded participants who did not consent for the data linkage (n=1040, 13%). Also, we excluded those who had a pre-existing CVD condition (n=2683, 37%), either self-reported in ELSA or identified by primary and secondary diagnoses in the APC data. This left us with a sample of 4587 participants. Of these, 16% had an AR-CVD (only 6% CVD death) and 7% died from non-CVD causes by January 2018.

#### Loneliness

Loneliness was measured using the three-item subscale from the revised University of California, Los Angeles loneliness scale, a validated and widely used tool.^17^ The questions include: (1) how often do you feel lack companionship? (2) how often do you feel isolated from others? (3) how often do you feel left out? Responses to each question were scored on a 3-point Likert scale ranging from hardly ever/never, to some of the time, to often. Using the sum score, we had a loneliness scale ranging from 3 to 9, with a higher score indicating increased loneliness. The distribution of loneliness was positively skewed (skewness=1.25).

#### Social isolation

The measure of social isolation was adapted from the study by Shankar *et al*.^17^ Participants were assigned one point for each of the following seven items: living alone, having less than monthly contact with children, relatives and friends, not belonging to any social organisation or club, not working and not volunteering. Different from the study by Shankar *et al*,^17^ we considered only meeting in-person and speaking on the telephone as the questions on writing/emailing had low factor loadings when tested in factor analysis. While the original scale included five items, we added working and volunteering to take into account any social contact through the network of colleagues.^15^ The social isolation scale ranged from 0 to 7, with the distribution being slightly skewed (skewness=0.31).

#### Covariates

We identified covariates using directed acyclic graphs, a graphic tool to better understand confounding bias (see the online supplementary file). These covariates included sociodemographic variables, including age groups (50–59, 60–69, 70–79, 80+ years), sex (male vs female), ethnicity (white vs non-white) and socioeconomic status generated using principal component analysis based on education, social class and household wealth (see the online supplementary file).

10.1136/heartjnl-2020-316614.supp1 Supplementary data

In the analyses, we also considered risks factors that could act as confounders but equally could lie on the causal pathway between loneliness, social isolation and CVD. We constructed a risk index to avoid multicollinearity, taking into account a wide range of modifiable risks factors that were well established in the literature, including obesity, high cholesterol, hypertension, diabetes, smoking, diet, physical activity, abnormal sleep and depression.^6 7 10^ The index was generated from confirmatory factor analysis (CFA), with a higher value indicating a higher CVD risk (see the online supplementary file).

### Statistical analysis

We used survival analysis to model the time from the baseline until the first CVD event or the end of the study. In the analysis of SR-CVD, participants who had not reported any CVD diagnosis were censored at the time of their last interview. The mean follow-up was 6.7 years. In the analysis of AR-CVD, participants were censored on 31 January 2018, providing a mean follow-up of 8.3 years. While no death could occur before the last interview in the SR cohort, the AR cohort members could be censored due to death. Mortality from a non-CVD cause was considered as a competing risk event. In this study, we adopted the Cox cause-specific hazards approach under which participants who had a competing event were removed from later risk sets.

The proportional hazards (PH) assumption of the Cox models was checked using both graphical diagnostics and statistical tests. There was no evidence the PH assumption was violated for the Cox models for SR-CVD, or the competing risks models for AR-CVD.

To address potential biases due to missing data, multiple imputation by chained equations was implemented. The number of imputation (n=30) was based on the percentage of missingness in the raw data (25%–26%). We included the Nelson-Aalen estimate of cumulative hazard alongside an event indicator as auxiliary variables in the multiple imputation model. The indices using CFA were generated in R V.3.5.1, and all further analyses were carried out using Stata V.15.

### Patient and public involvement

In designing this observational study, we consulted with clinicians and community workers who have experience in working with people who are lonely and/or socially isolated. Their comments were used to inform the study design and discussions.

## Results

### Study sample

**Figure 2:**
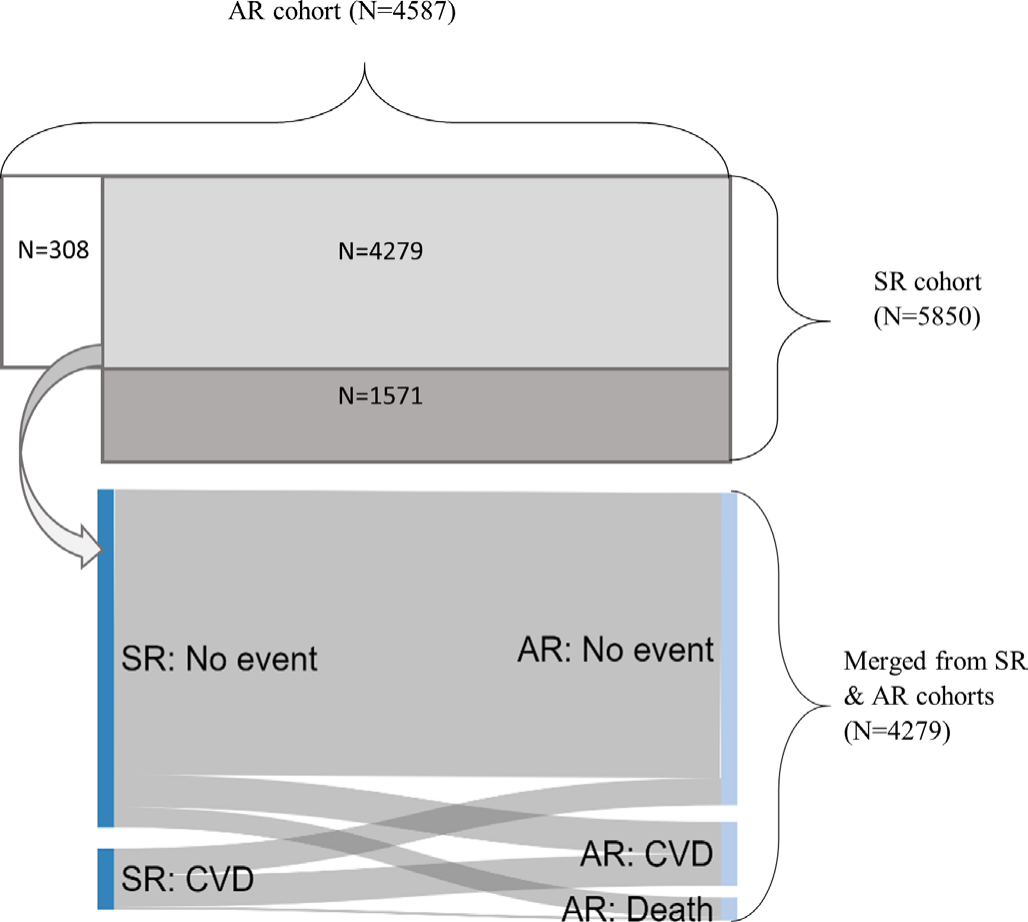
Comparison between the SR and AR cohorts (the upper panel showing how the participant overlap between cohorts, the lower panel comparing event differences for the merged cohort). AR, administrative record; CVD, cardiovascular disease; SR, self-reported.

The SR and AR cohorts largely shared the same participants as they were derived from the same data source. As illustrated in figure 2, there were 4279 participants who were members of both cohorts, making up 93% of the AR and 73% of the SR cohort. There were 308 participants (7%) from the AR cohort who were not in the SR cohort. These were individuals who did not have a follow-up interview after the baseline but whose data were still available through data linkage. There were 1571 participants (27%) who were included in the SR cohort but not in the AR cohort. They were mostly participants who did not consent for data linkage.

As shown in figure 2, there were some inconsistencies between two sources for merged participants. Ten per cent of participants who did not report any newly diagnosed CVD in ELSA had a CVD-related hospital admission. This could be under-reporting or due to the difference in follow-up periods between two cohorts. Furthermore, only 51% of those who had a CVD diagnosis had an AR-CVD. Among people who had an AR-CVD, only 49% had a self-reported diagnosis.

Table 1 shows the characteristics of participants in the SR and AR cohorts. Generally speaking, these two cohorts had similar characteristics.

**Table 1:** Characteristics of participants in the self-reported (SR) cohort and the cohort with administrative record (AR)

	SR cohort	AR cohort
Loneliness	4.14 (1.51)	4.09 (1.48)
Social isolation	2.33 (1.32)	2.30 (1.32)
Female	56.85%	56.22%
Non-white*	2.44%	1.98%
Age (years)		
50–59	34.19%	35.12%
60–69	39.20%	39.79%
70–79	20.75%	19.42%
80+	5.86%	5.67%
Socioeconomic status	0.09 (1.33)	0.13 (1.33)
Cardiovascular risk	−0.02 (0.39)	−0.05 (0.38)

Results based on 30 multiply imputed datasets.

*Asian, black, mixed and other ethnic groups.

### Main findings

**Figure 3:**
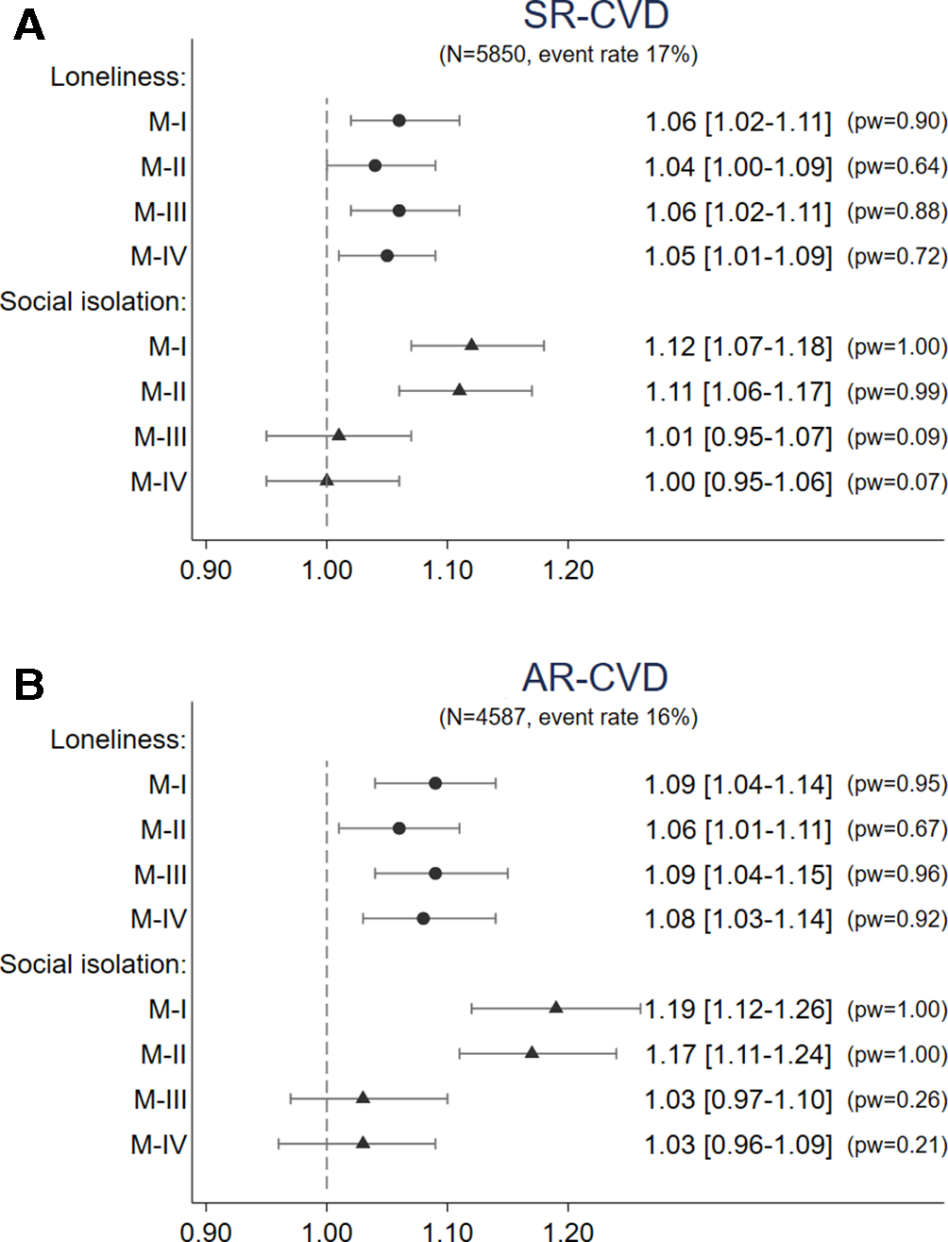
(A) HR, 95% CI and power from Cox models for SR-CVD and AR-CVD (model I: unadjusted model including only loneliness OR social isolation; model II: loneliness+social isolation; model III: model II+demographics; model IV: model III+CVD risk). Results based on 30 multiply imputed datasets. (B) HR, 95% CI and power from Cox models for SR-CVD and AR-CVD (model I: unadjusted model including only loneliness OR social isolation; model II: loneliness+social isolation; model III: model II+demographics; model IV: model III+CVD risk). Results based on 30 multiply imputed datasets. AR, administrative record; CVD, cardiovascular disease; SR, self-reported.

Figure 3A presents the estimates for loneliness and social isolation from the survival analysis of SR-CVD. According to the unadjusted model (model I), people who were socially isolated had a significantly higher hazard of CVD (HR: 1.12, 95% CI: 1.07 to 1.18). This effect was attenuated after controlling for loneliness and potential confounders. Loneliness (HR: 1.06, 95% CI: 1.02 to 1.11), however, was a persistent predictor of CVD independent of sociodemographic factors and social isolation (model III). The association between loneliness and CVD held even after controlling for baseline CVD risk (model IV). One point increase in loneliness was associated with a 5% increase in the hazard of CVD (95% CI: 1.01 to 1.09), meaning that the hazard was 30% higher for people with the highest loneliness score than the lowest.

The results for AR-CVD are reported in figure 3B. Again, social isolation was associated with the risk of AR-CVD, but the association was not independent of loneliness or sociodemographics. However, there was a strong and persistent association between loneliness and the risk of CVD events, which was not fully explained by the differences in CVD risks. One point increase in loneliness was associated with an 8% higher hazard of CVD hospital admissions, independent of CVD risks (95% CI: 1.03 to 1.14). The hazard for people with the highest loneliness scores were 48% higher than those with the lowest.

We tested whether the findings differed by gender, age, socioeconomic status or CVD risk, but no evidence of moderation effect was found. Sensitivity analyses excluding angina and shortening the AR-CVD follow-up time in line with the follow-up time for SR-CVD did not affect the results.

## Discussion

Using a large nationally representative sample of adults aged 50+ years, this study found that loneliness but not social isolation, is independently associated with higher risk of onset CVD and CVD-related hospital admissions. These findings echo a previous study also using ELSA data,^15^ but crucially they build on the previous work by linking ELSA data to hospital records, enabling the analysis of a full range of CVDs from hospital diagnosis. Therefore, our results confirm the finding that loneliness is associated with higher CVD risk in an updated sample, and shed light on health service utilisation related to CVD and loneliness, but more research is needed to clarify other metrics such as number of admissions, length of hospital stay or other forms primary and secondary healthcare utilisation.

A number of possible underlying mechanisms have been suggested to explain the relationship between loneliness and health. A common explanation is that loneliness influences health via other conventional risk factors, such as health behaviours, physical and mental health conditions.^18^ There is a wealth of empirical evidence showing that loneliness predicts physical inactivity,^19^ alcohol abuse,^20^ smoking,^21^ obesity,^22^ high blood pressure^23^ and depression.^24^ Our analyses have shown a significant association between loneliness and CVD even after accounting for these risk factors that are arguably on the causal pathway. This, therefore, suggests that loneliness may influence CVD events through other channels.

Our finding of a relationship with CVD for loneliness but not social isolation suggests that the subjective aspects of social connections are more important in relation to CVD than the objective factor as to whether an individual is socially isolated. This matches previous studies on all-cause mortality and depression.^25 26^ In considering how subjective assessment of social connections might affect CVD incidence, two main theories have been proposed. First, it is possible that loneliness influences CVD through increasing psychological stress.^27^ There is a large literature on stress as a risk factor for CVD.^9 28^ While our results were independent of hypertension, psychological stress does not always relate to blood pressure.^29^ Second, it is possible that loneliness affects CVD directly through inflammatory pathways. These and other pathways remain to be explored in future studies.

One of the main strengths of our study is the use of data from a large-scale nationally representative survey, allowing for generalisability. Moreover, by linking the survey data to administrative records, we were able to look at both self-reported CVD diagnosis and hospital admissions related to CVD and to cross-validate our own findings. Finally, our study employs a longitudinal design with a follow-up period up to nearly a decade, which reduces the possibility of reverse causality. However, we are aware a longitudinal design is not sufficient to establish causality. We could not completely rule out the possibility of residual confounding due to the omission of confounders. Any causal inference from these findings should be made with caution. For the analysis of self-reported CVD diagnosis, when diagnosis dates were unavailable, interview dates were used as a proxy, which arguably might overestimate the survival time. However, given the same method was applied to all participants without a diagnosis date, it is unlikely it would bias the estimates systematically. It is also reassuring that the analysis of hospital admission gives consistent results where the exact date of diagnosis were available.

Overall, our study provides strong evidence that loneliness is related to an increased risk of CVD and related hospitalisations. This suggests the importance of acknowledging loneliness as an additional psychosocial risk factor for CVD. In recent years, the NHS is evolving towards a more holistic approach of providing care, by implementing integrated, personalised care. A key component of this process is social prescribing, a way of linking patients in primary care with community resources.^30^ The findings presented here suggest the potential value of referring individuals at high risk of CVD who are lonely to social activities. Future studies are encouraged to explore whether social prescribing could help reducing the incidence of cardiovascular disease among adults approaching old age.

**Key questions**

**What is already known on this subject?**

- Loneliness and social isolation are risk factors for all-cause and cardiovascular disease (CVD)-specific mortality.

**What might this study add?**

- People with the highest level of loneliness were at an increased risk of 30%–48% for self-reported CVD diagnosis and CVD-related hospital admissions, controlling for social demographic, objective social factors and CVD risks.

**How might this impact on clinical practice?**

- Loneliness should be included as a psychosocial risk factor of CVD in clinical practice.
