# Supplementary material for: Longitudinal associations between loneliness, social isolation and cardiovascular events
Source: Heart. 2020 May 27;106(18):1394–9. doi: 10.1136/heartjnl-2020-316614 (PMC7497558; doi:10.1136/heartjnl-2020-316614)
Supplement: Supplementary data [file heartjnl-2020-316614supp001.pdf]

Supplementary Material

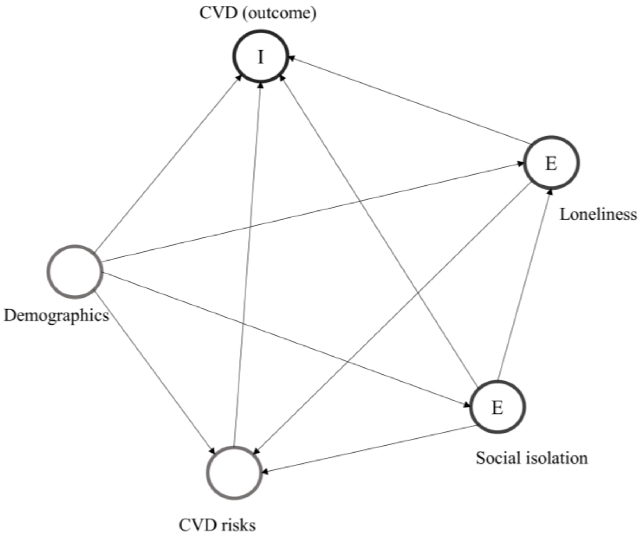

Figure S1 Directed acyclic graph (DAG)

Socioeconomic Status Index (higher value, higher SES)

Educational qualification: (1-degree or equivalent, 2-higher education below degree, 3-GCE A levels, NVQ3 or equivalent, 4-GCE O levels, NVQ2 or equivalent, 5-CSE/NVQ1 or equivalent, 6-Other, 7-No qualification)

Social class: (1-managerial and professional occupations, 2-intermediate occupations, 3-small employers and own account workers, 4-lower supervisory and technical occupations, 5-semi-routine and routine occupations, 6-never worked, unemployment or other)

Total net (non-pension) wealth (in quintiles, lower value wealthier)

Table S1 Results from the principle component analysis

Component	Eigenvalue	Difference	Proportion	Cumulative
1	1.81	1.13	0.60	0.60
2	0.67	0.15	0.22	0.83
3	0.52	.	0.17	1.00

CVD Risk Index

Hypertension (HYP) was defined as having a self-reported diagnosis of hypertension or having a systolic blood pressure $\geq$ 140 mm Hg or diastolic blood pressure $\geq$ 90 mm Hg.[1]

Cholesterol risk (CHO) was derived based on total cholesterol to HDL-cholesterol (TC/HDL-C) ratio, which was defined as > 4 for men and >3.5 for women.[2]

Bu F, *et al. Heart* 2020; 106:1394–1399. doi: 10.1136/heartjnl-2020-316614

Diabetes (DIA) was defined as having self-reported diagnosis of diabetes or a fasting glucose level ≥7 mmol/l.[3]

Obesity (OBE) was defined as having a BMI ≥ 30 in line with the WHO definition.

Abdominal obesity (ABD) was defined as having a waist circumference ≥ 102 cm for men and ≥ 88 cm for women.[4]

Depression (DEP) were measured using the eight-item Centre for Epidemiological Studies-Depression (CES-D) scale, in addition to reported physician diagnoses of clinical depression. We derived a summary score by adding binary responses to all 8 questions. The cut point of 4 was used for defining the risk of serious depressive symptoms as validated in previous studies.[5]

Abnormal sleep (SLE) was defined as ≤ 5 hours or ≥ 9 hours of sleeping.[6]

Smoking (SMO) was coded as non-smoker, ex-smoker and current smoker.

Poor diet (DIE) was defined as having < 5 portions of fruits and vegetables daily which is the public health recommended intake in the UK.

Physical inactivity (INA) was defined as people who exercised less than weekly at either vigorous (e.g. running, swimming, cycling, aerobics and tennis) or moderate levels (gardening, walking, dancing etc).

The confirmatory factor analysis (CFA) model based on these risk indicators is presented in Figure S1. As all indicators were coded as binary variables, the CFA model was fitted using diagonally weighted least squares (DWLS) indicators. The root mean square error of approximation (RMSEA) had a value of 0.03, showing a good fit[7]. This was also confirmed by the comparative fit index (CFI=0.99), and Tucker–Lewis index (TLI=0.98).

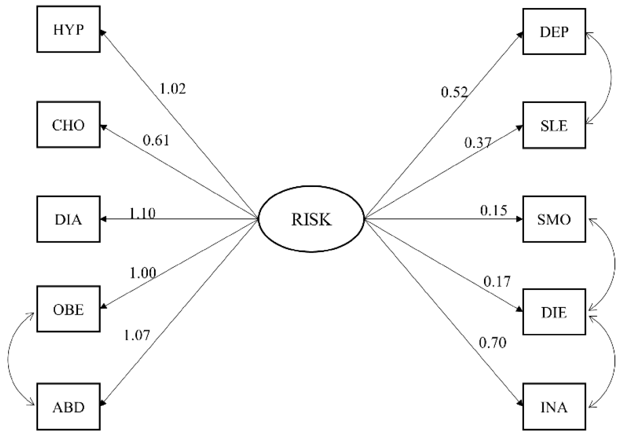

Figure S2 CFA diagram and factor loadings

References

1. Mancia G, Sega R, Milesi C, et al. Blood-pressure control in the hypertensive population. *The Lancet* 1997;349(9050):454-57. doi: https://doi.org/10.1016/S0140-6736(96)07099-7
2. Millán J, Pintó X, Muñoz A, et al. Lipoprotein ratios: Physiological significance and clinical usefulness in cardiovascular prevention. *Vasc Health Risk Manag* 2009;5:757-65. [published Online First: 09/18]
3. The Expert Committee on the Diagnosis and Classification of Diabetes Mellitus. Report of the Expert Committee on the Diagnosis and Classification of Diabetes Mellitus. *Diabetes Care* 2003;26(suppl 1):s5-s20. doi: 10.2337/diacare.26.2007.S5
4. McGee S. Evidence-Based Physical Diagnosis 4ed. Philadelphia: ELSEVIER 2018.
5. Han B. Depressive Symptoms and Self-Rated Health in Community-Dwelling Older Adults: A Longitudinal Study. *Journal of the American Geriatrics Society* 2002;50(9):1549-56. doi: 10.1046/j.1532-5415.2002.50411.x
6. Min Y, Slattum PW. Poor Sleep and Risk of Falls in Community-Dwelling Older Adults: A Systematic Review. *Journal of Applied Gerontology* 2016;37(9):1059-84. doi: 10.1177/0733464816681149
7. Hooper D, Coughlan J, Mullen MR. Structural equation modelling: Guidelines for determining model fit. *Electronic journal of business research methods* 2008;6(1):53-60.

Bu F, *et al. Heart* 2020; 106:1394–1399. doi: 10.1136/heartjnl-2020-316614
